# Perceived Vaccination Knowledge and Self-Reported Uptake in a Nationwide Heart Failure Telemonitoring Cohort in France

**DOI:** 10.3390/jcm15176933

**Published:** 2026-09-07

**Authors:** Patrick Jourdain, Rémi Esser, Nicolas Girerd, Abdelhakim Hacil, Nathalie Berkane, Hélène Benchimol, Franck Albert, Annabelle Jagu, François Picard, Mihaela Ionescu, Wafa Ben Ghezala, Nicolas Pages, Sophie Nisse-Durgeat, Olivier Hanon

**Affiliations:** 1 Ramsay Santé Medical Department, Ramsay Santé, 75017 Paris, France; 2 Université Paris-Saclay, AP-HP, Hôpital Bicêtre, 94270 Le Kremlin-Bicêtre, France; 3 Department of Cardiogeriatrics, Hôpital La Porte Verte, 78000 Versailles, France; remi.esser@lpv.univi.fr; 4 Centre d’Investigations Cliniques Plurithématique 1433 and Inserm U1116, CHRU Nancy, FCRIN INI-CRCT (Cardio-vascular and Renal Clinical Trialists), Université de Lorraine, 54500 Nancy, France; 5 Geriatrics Department, Broca Hospital, Assistance Publique–Hôpitaux De Paris, 75013 Paris, France; 6 Cardiology Department, Simone Veil Hospital, 06150 Cannes, France; 7 Cardiology Department, Saintonge Hospital, 17000 Saintes, France; 8 Cardiology Department, Chartres Hospital, 28000 Chartres, France; 9 Cardiology Department, Groupe Hospitalier Paris Saint-Joseph, 75014 Paris, France; 10 Cardiology Department, Haut-Lévêque Hospital, Bordeaux University Hospital, 33600 Pessac, France; 11 Cardiology Department, Avignon Hospital, 84000 Avignon, France; mionescu@ch-avignon.fr; 12 Geriatrics Department, Les Magnolias Hospital, 91160 Ballainvilliers, France; 13 NP Medical, 33000 Bordeaux, France; 14 Gérontopôle d’Île-De-France, Université Paris Cité, 75006 Paris, France

**Keywords:** heart failure, vaccination, telemonitoring, vaccine uptake, cardiovascular prevention, influenza, pneumococcal vaccination

## Abstract

**Background/Objectives**: Patients with heart failure (HF) are vulnerable to vaccine-preventable infections, yet vaccination data remain limited. We assessed perceived vaccination knowledge, attitudes, self-reported vaccine receipt, and variation by age, sex, region, and delivery pathway among digitally connected patients with HF enrolled in a nationwide telemonitoring program. **Methods**: This cross-sectional electronic survey was conducted from 5 to 28 November 2025, during the ongoing 2025–2026 influenza and COVID-19 vaccination campaigns, among app-connected Satelia^®^ Cardio participants across France. Of 4856 invited app-connected patients, 1100 completed the questionnaire (response rate: 22.7%). Uptake of seasonal influenza and COVID-19 booster vaccination for the 2025–2026 season and lifetime receipt of pneumococcal, tetanus–diphtheria–pertussis–poliomyelitis (Tdap-polio), and herpes zoster vaccines were analyzed using Wilson 95% confidence intervals (CIs), age-adjusted logistic regression, and regional models adjusted for age and sex. **Results**: Respondents had a mean age of 71.5 years (SD: 12.6), and 718 (65.3%) were men. Overall, 87.5% considered vaccination useful, 96.0% knew where to be vaccinated, and 55.1% wanted telemonitoring-based reminders. Current-season reported vaccination was 62.2% for influenza and 31.4% for the COVID-19 booster, whereas lifetime self-reported receipt was 38.5% for Tdap-polio, 34.5% for pneumococcal vaccination, and 11.5% for herpes zoster. Influenza and COVID-19 uptake increased with age, whereas pneumococcal uptake peaked at 65–74 years. Pneumococcal receipt was higher in women than men (42.2% vs. 30.4%; age-adjusted odds ratio [aOR], 1.67; 95% confidence interval [CI], 1.29–2.17; *p* < 0.001). Community pharmacies delivered most influenza and COVID-19 vaccinations. **Conclusions**: Vaccination attitudes were favorable, while current-season vaccination and lifetime-reported vaccine receipt showed distinct patterns across age groups. These descriptive findings identify potential target groups for future telemonitoring-based preventive interventions, whose effectiveness requires prospective evaluation.

## 1. Introduction

Heart failure (HF) affects more than 1 million people in France, with a prevalence of 1.7% and a substantial and growing hospital burden [1]. Patients with HF are particularly vulnerable to respiratory and invasive infections, which can precipitate acute decompensation, hospitalization, and major adverse cardiovascular events [2,3,4], a vulnerability compounded in this predominantly older population by the age-related decline in immune competence [5]. Influenza, pneumococcal, and SARS-CoV-2 vaccination have each been associated with reduced cardiovascular morbidity and mortality in cardiovascular populations [2,4,6,7,8], herpes zoster vaccination has been linked to lower rates of stroke and myocardial infarction in adults with chronic conditions [9], and the 2025 European Society of Cardiology (ESC) clinical consensus statement now positions vaccination as a foundational pillar of cardiovascular prevention [2,4,6]. Despite these recommendations, adult vaccination coverage remains suboptimal and unevenly documented in HF, and few contemporary studies describe coverage across the full panel of recommended adult vaccines, its variation across the age range, or the pathways through which patients are vaccinated.

Remote HF telemonitoring programs, now reimbursed and widely deployed in France, maintain structured contact with geographically dispersed patients and could support preventive-care assessment and personalized prompts. Such programs have also been associated with lower mortality and hospitalization [10,11]. However, vaccination patterns among telemonitored patients with HF remain poorly characterized. We therefore conducted a cross-sectional electronic survey within a nationwide HF telemonitoring program to describe perceived vaccination knowledge, attitudes, self-reported vaccine receipt, and variation by age, sex, region, and delivery pathway.

## 2. Materials and Methods

### 2.1. Study Design and Participants

We conducted a cross-sectional, self-administered electronic survey from 5 November to 28 November 2025, among adults enrolled in the Satelia^®^ Cardio (NP Medical, Bordeaux, France) HF telemonitoring program across metropolitan and overseas France. The survey was conducted during the ongoing 2025–2026 influenza and COVID-19 vaccination campaigns rather than after campaign completion. Eligible participants were app-connected patients—that is, patients who interact with the telemonitoring platform through the mobile/web application rather than exclusively by telephone—because the questionnaire was administered electronically through the application. Patients followed exclusively by telephone were therefore not eligible for the survey and are not represented in the study population. Of 4856 invited app-connected patients, 1100 completed the questionnaire, corresponding to a response rate of 22.7%. Individual-level demographic and clinical characteristics of nonresponders were unavailable; therefore, respondent–nonrespondent comparisons could not be performed. The response rate was calculated as the number of completed questionnaires divided by the number of eligible app-connected patients invited. Participation was voluntary, and the analyzed dataset was pseudonymized before analysis. Reporting was informed by the American Association for Public Opinion Research (AAPOR) survey principles and the Strengthening the Reporting of Observational Studies in Epidemiology (STROBE) recommendations [12].

### 2.2. Questionnaire and Measures

The questionnaire captured age, self-reported sex (woman, man, or prefer not to specify), and postal code, and assessed (1) self-reported knowledge of age-recommended vaccinations, (2) perceived usefulness of vaccination, (3) self-reported knowledge of where to be vaccinated, (4) prior difficulty accessing vaccination, (5) interest in reminders from the telemonitoring team, and (6) intention to vaccinate if recommended by a physician. The question regarding age-recommended vaccinations assessed participants’ perceived knowledge and did not constitute an objective test of vaccination knowledge. Participants reported receipt of 5 adult vaccines: seasonal influenza and the seasonal COVID-19 booster for the 2025–2026 season, and lifetime receipt (“ever received”) of pneumococcal, tetanus–diphtheria–pertussis–poliomyelitis (Tdap-polio), and herpes zoster vaccines. For each vaccine reported as received, participants also indicated the category of vaccinating provider (primary care physician, pharmacist, nurse, vaccination center, geriatrician, or cardiologist) (Supplementary Questionnaire S1). Lifetime receipt did not establish whether an age-specific vaccination schedule had been completed or remained up to date.

For the primary vaccine-receipt analyses, participants answering “Yes” were classified as reporting vaccine receipt. Responses of “No” and “I don’t know” were retained as distinct response categories but were both included in the denominator of the primary analysis as not reporting receipt. Because “No” and “I don’t know” represent conceptually different responses, sensitivity analyses excluded “I don’t know” responses and recalculated vaccine receipt among participants providing a definite “Yes” or “No” response. For binary knowledge and attitude outcomes, only “Yes” responses were classified as affirmative; “No,” “I don’t know,” and “A little,” where applicable, were classified as nonaffirmative. Individual vaccine indications, contraindications, prior adverse reactions, and schedule eligibility were not assessed in the survey. The analytical dataset did not include detailed HF-related clinical characteristics such as HF phenotype, left ventricular ejection fraction, NYHA functional class, etiology, frailty, comorbidities, recent hospitalization, or current HF therapy.

### 2.3. Ethical Considerations

This study was conducted in accordance with the applicable French regulatory requirements governing noninterventional surveys using pseudonymized health data. Under these requirements, review by a Comité de Protection des Personnes (CPP) was not required. Participants had previously consented to the use of their data within the telemonitoring program and voluntarily agreed to participate by completing and electronically submitting the questionnaire. No financial compensation was provided.

### 2.4. Statistical Analysis

Categorical variables are summarized as number (percentage) [n (%)]. In the primary analysis, self-reported vaccine receipt was defined as the proportion answering “Yes” among all respondents, with 95% CIs calculated using the Wilson score method [13]. Uptake was described across 5 age strata (18–49, 50–64, 65–74, 75–84, and ≥85 years) (Table S1) and 3 broad groups (<65, 65–84, and ≥85 years) (Table S2). Perceived vaccination knowledge, attitudes, and reminder interest were summarized across the 5 age strata (Table S3). Age was also modeled continuously using restricted cubic splines with 4 knots at the 5th, 35th, 65th, and 95th percentiles (49, 67, 78, and 89 years) [14]. Spline models were adjusted for sex but not region because within-region samples were insufficient for stable estimation. For each vaccine, likelihood-ratio tests assessed overall age association (spline vs. sex-only model) and nonlinearity (spline vs. linear-age model). The overall age test assessed any association, whereas the nonlinearity test assessed deviation from a linear relationship. Predicted probabilities and 95% CIs were obtained by marginal standardization over the observed sex distribution (Figure S1; Table S4). Sex differences were tested with χ^2^ tests and age-adjusted logistic regression. Respondents with missing sex were retained in overall analyses but excluded from sex-stratified and adjusted analyses. As a sensitivity analysis, “I don’t know” responses were treated as uncertain vaccination status and excluded from the denominator for each vaccine. Vaccine receipt was then recalculated among participants with definite “Yes” or “No” responses (Table S8). The main age-related analyses and age-adjusted sex associations were repeated using these restricted denominators (Table S9). Sex-by-age interactions were tested, with the 5 interaction *p*-values adjusted using the Benjamini–Hochberg procedure (Table S5). Respondents were assigned to French administrative regions from postal codes. Regions with at least 10 respondents were retained for the primary regional analysis, and regional heterogeneity was tested using likelihood-ratio tests comparing models with and without region, adjusted for age and sex. As a sensitivity analysis, these tests were repeated after excluding regions with fewer than 20 respondents (Table S10). Vaccinating-provider distributions were compared by sex among vaccinated respondents (χ^2^). No imputation was performed. Age had no missing data; “I don’t know” vaccination responses were handled as described above rather than considered missing in the primary analysis. Secondary and exploratory *p*-values should be interpreted descriptively because of multiple comparisons. Regional analyses were considered exploratory *p*-values because adjustment was limited to age and sex, relevant clinical and socioeconomic covariates were unavailable, and several regions had small sample sizes. Two-sided *p* < 0.05 defined statistical significance. Analyses were performed using Python version 3.11.9 (Python Software Foundation, Wilmington, DE, USA) and statsmodels version 0.14.4.

### 2.5. Use of Generative Artificial Intelligence

During the preparation of this manuscript, the authors used ChatGPT (GPT-5.6 Thinking; OpenAI, San Francisco, CA, USA) for English-language editing and manuscript formatting. The tool was not used for data collection, statistical analysis, or generation of study results. The authors reviewed and edited the output and take full responsibility for the content of this publication.

## 3. Results

### 3.1. Participants

Of 4856 app-connected adults invited between 5 November and 28 November 2025, 1100 (22.7%) completed the questionnaire (mean [SD] age, 71.5 [12.6] years; median, 73 years; range, 25–100 years). Of these, 718 (65.3%) were men, 379 (34.5%) were women, and 3 (0.3%) did not specify sex. Age distribution and characteristics are shown in Table 1.

### 3.2. Perceived Knowledge and Attitudes

Attitudes toward vaccination were broadly favorable (Table 1): 963 patients (87.5%) considered vaccination useful to protect their health, 1056 (96.0%) knew where to be vaccinated, and only 19 (1.7%) reported prior difficulty accessing vaccination. Self-reported knowledge of age-recommended vaccines was reported by 825 participants (75.0%), 882 (80.2%) would vaccinate if their physician recommended it, and 606 (55.1%) were interested in receiving reminders from the telemonitoring team.

### 3.3. Self-Reported Vaccine Receipt

Current-season self-reported vaccination was 62.2% (95% CI, 59.3–65.0%) for influenza and 31.4% (95% CI, 28.7–34.2%) for the COVID-19 booster. Lifetime self-reported receipt was 38.5% (95% CI, 35.7–41.5%) for Tdap-polio, 34.5% (95% CI, 31.7–37.3%) for pneumococcal vaccination, and 11.5% (95% CI, 9.8–13.6%) for herpes zoster. Among patients who reported being vaccinated against influenza every year, 22.3% had not yet reported current-season influenza vaccination at the time of the survey; the corresponding proportion not yet reporting a current-season COVID-19 booster was 32.0%.

“I don’t know” responses accounted for 10 (0.9%) responses for influenza, 10 (0.9%) for the COVID-19 booster, 102 (9.3%) for pneumococcal vaccination, 164 (14.9%) for Tdap-polio, and 70 (6.4%) for herpes zoster. After exclusion of these responses, self-reported vaccine receipt was 62.8%, 31.7%, 38.0%, 45.3%, and 12.3%, respectively (Table S8). In the corresponding sensitivity analyses, overall age associations remained for influenza, the COVID-19 booster, pneumococcal vaccination, and herpes zoster, and an overall age association emerged for Tdap-polio (*p* = 0.02). Evidence of nonlinearity for the COVID-19 booster was attenuated (*p* = 0.10), whereas nonlinearity remained evident for pneumococcal and herpes zoster vaccination. The age-adjusted association between female sex and higher pneumococcal receipt remained present (adjusted OR: 1.62; 95% CI, 1.24–2.11; *p* < 0.001), while no significant sex association was observed for the other vaccines (Table S9).

### 3.4. Age Patterns

**Figure 1:**
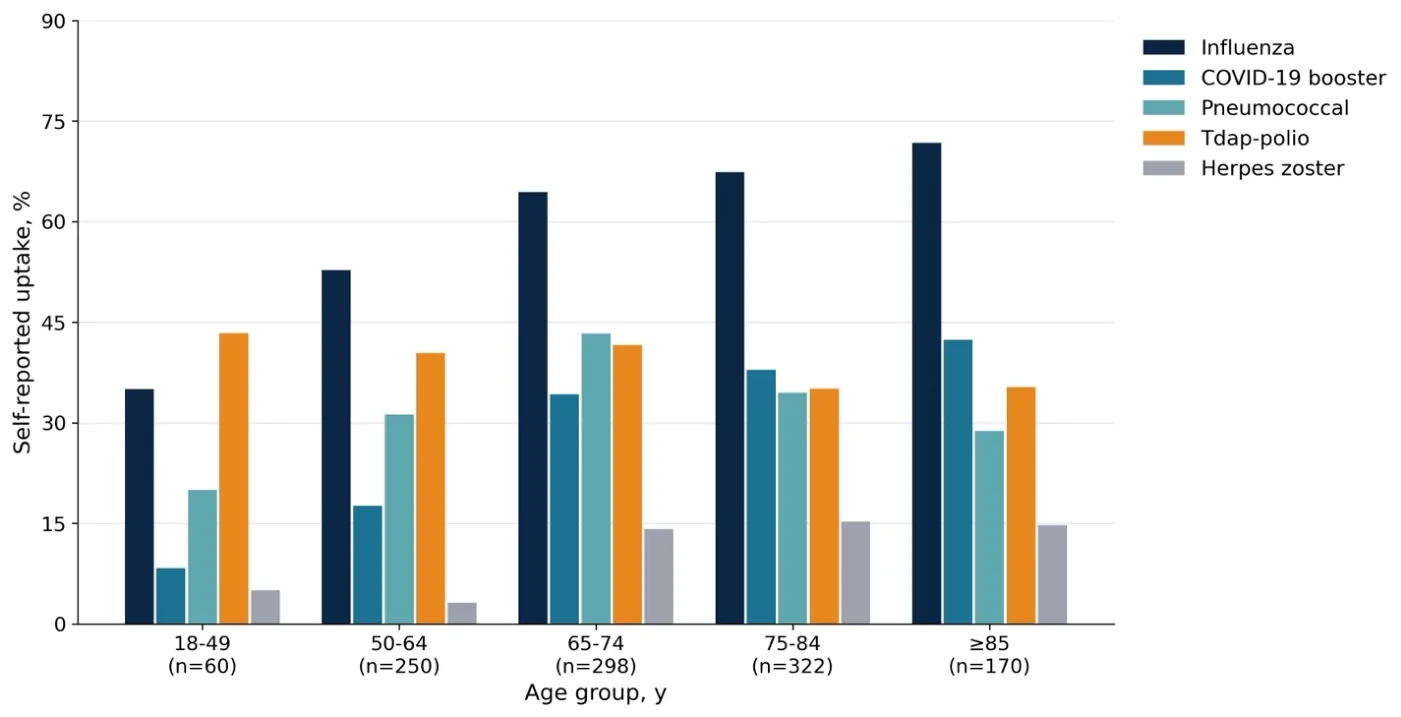
Self-Reported Seasonal Vaccination and Lifetime Vaccine Receipt by Age Group.

Self-reported vaccine receipt showed distinct age patterns (Figure 1; Table S1; Figure S1). Influenza increased from 35.0% at ages 18–49 years to 71.8% at 85 years or older, and the COVID-19 booster from 8.3% to 42.4%. Pneumococcal uptake peaked at ages 65–74 years (43.3%) and was lower in the youngest (20.0%) and oldest (28.8%) groups. Tdap-polio uptake was relatively stable, whereas herpes zoster remained low (≤5% before age 65 years and approximately 14–15% thereafter). Sex-adjusted spline models showed overall age associations for influenza, COVID-19, pneumococcal, and herpes zoster vaccination (all *p* ≤ 0.03), but not Tdap-polio (*p* = 0.47). Nonlinearity was present for pneumococcal (*p* = 0.02), herpes zoster (*p* = 0.001), and COVID-19 vaccination (*p* = 0.04), but not influenza (*p* = 0.96) or Tdap-polio (*p* = 0.77). Pneumococcal uptake peaked at age 71 years (39.9%; 95% CI, 35.1–44.9%). Estimates at ages 25 and 100 years were imprecise because of sparse data.

Self-reported current-season vaccination and lifetime vaccine receipt among 1100 patients with heart failure enrolled in a nationwide telemonitoring program, according to age group. Influenza and COVID-19 booster uptake refer to the 2025–2026 season; pneumococcal, tetanus–diphtheria–pertussis–poliomyelitis (Tdap-polio) booster, and herpes zoster uptake refer to ever-reported receipt. Age-group sample sizes were 60 for 18–49 years, 250 for 50–64 years, 298 for 65–74 years, 322 for 75–84 years, and 170 for 85 years or older.

### 3.5. Age-Group Comparison

To provide a clinically interpretable descriptive summary complementary to the continuous-age analysis, we compared 3 broad age groups: <65 years (n = 310), 65–84 years (n = 620), and ≥85 years (n = 170) (Table S2). Influenza (49.4%, 66.0%, 71.8%) and the COVID-19 booster (15.8%, 36.1%, 42.4%) increased progressively across these groups. Pneumococcal uptake followed a non-monotonic pattern (29.0%, 38.7%, and 28.8%, respectively): patients ≥ 85 years did not extend the upward trend observed from <65 to 65–84 years, and their uptake (28.8%) was closer to that of patients < 65 years than to the pooled ≥65 years estimate (36.6%), indicating that a simple < 65-vs.-≥65 dichotomization would have obscured this pattern. The Tdap-polio booster was comparatively stable across groups (41.0%, 38.2%, 35.3%).

### 3.6. Oldest Patients

Patients aged 85 years or older (n = 170) reported particularly favorable attitudes toward vaccination: 92.9% considered vaccination useful (vs. 82.6% in patients < 65 years), 78.8% reported knowing the vaccines recommended for their age, and 87.6% would vaccinate on physician advice (vs. 68.4% in patients < 65 years); none reported prior difficulty accessing vaccination (Table S3). Despite this, ever-reported pneumococcal receipt in this group (28.8%) was lower than in patients aged 65–74 years (43.3%), and interest in reminders from the telemonitoring team was the lowest of any age stratum (47.6%, vs. 54–59% in other groups). Among vaccinated patients, the share of influenza vaccinations delivered by a nurse rather than a pharmacist rose from 17.0% in patients < 65 years to 33.6% in patients aged 85 years or older, while the pharmacy share fell from 70.6% to 55.7% (Table S6). This pattern may reflect greater reliance on nurse-delivered vaccination among the oldest patients, although the setting of vaccine administration (home visit, care facility, or outpatient) and mobility status were not recorded.

### 3.7. Sex Differences

Influenza uptake was similar by sex (women, 62.0%; men, 62.3%). Ever-reported pneumococcal receipt was higher in women than in men (42.2% vs. 30.4%; age-adjusted OR, 1.67; 95% CI, 1.29–2.17; *p* < 0.001) (Table 2). Women tended toward higher herpes zoster uptake (aOR, 1.42; *p* = 0.07) and lower COVID-19 booster uptake (aOR, 0.79; *p* = 0.09), without significant differences for Tdap-polio. In exploratory models testing a continuous age-by-sex interaction across the 5 vaccines (Table S5), nominal interaction *p*-values were 0.02 for pneumococcal vaccination (interaction OR, 0.98 per year; 95% CI, 0.96–1.00) and 0.03 for the Tdap-polio booster (interaction OR, 0.98 per year; 95% CI, 0.96–1.00), with no significant interaction for influenza, the COVID-19 booster, or herpes zoster (all *p* ≥ 0.57). After adjustment for multiple testing across the 5 interaction tests (Benjamini–Hochberg procedure), neither interaction remained significant (adjusted *p* = 0.06 for both), and these findings should be considered hypothesis-generating.

### 3.8. Regional Variation

**Figure 2:**
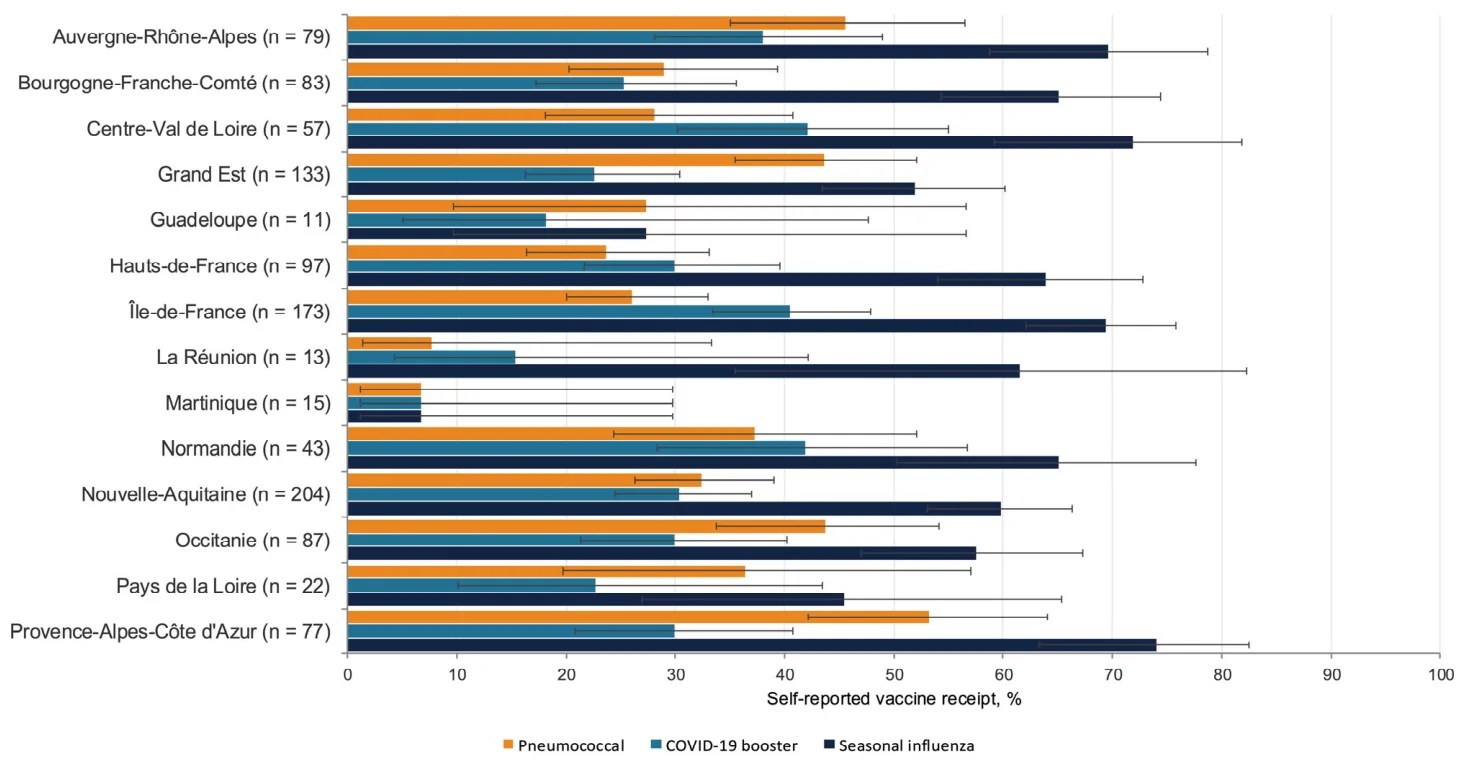
Exploratory Regional Estimates of Influenza, COVID-19 Booster, and Pneumococcal Vaccine Receipt.

Fourteen regions had at least 10 respondents (n = 11–204) (Table S7). Regional estimates showed substantial numerical variability, although several were based on small samples. In exploratory models adjusted for age and sex only, evidence of regional heterogeneity was observed for influenza (*p* < 0.001), pneumococcal vaccination (*p* < 0.001), and the COVID-19 booster (*p* = 0.02), but not for Tdap-polio (*p* = 0.86) or herpes zoster (*p* = 0.26). In a sensitivity analysis restricted to the 11 regions with at least 20 respondents (n = 1055), evidence of regional heterogeneity remained for influenza (*p* = 0.04), the COVID-19 booster (*p* = 0.03), and pneumococcal vaccination (*p* < 0.001), but not for Tdap-polio (*p* = 0.87) or herpes zoster (*p* = 0.49) (Table S10). Given the small and non-population-based regional samples and limited covariate adjustment, these findings should not be interpreted as robust evidence of geographic disparities. Regional denominators and 95% confidence intervals are reported in Table S7 and Figure 2.

Self-reported current-season influenza and COVID-19 vaccination and lifetime-reported pneumococcal vaccine receipt across the 14 French regions with at least 10 respondents. Regional sample sizes are shown beside the region names, and error bars represent Wilson 95% confidence intervals. Regional comparisons were exploratory because several regional samples were small, the sample was not population-based, and analyses were adjusted only for age and sex.

### 3.9. Vaccination Pathways

Community pharmacies were the dominant vaccinating provider for influenza (66.4%), the COVID-19 booster (81.2%), pneumococcal vaccination (43.0%), and herpes zoster (59.1%), whereas the Tdap-polio booster was mainly delivered by general practitioners (GPs) (54.2%) (Table 3). Vaccination centers, geriatricians, and cardiologists contributed minimally. Provider distributions differed by sex for influenza (*p* = 0.03) and the COVID-19 booster (*p* = 0.004), with GP delivery more frequent in women and pharmacy delivery more frequent in men.

## 4. Discussion

In this geographically distributed cohort of digitally connected patients enrolled in a nationwide HF telemonitoring program, attitudes toward vaccination were generally favorable. Current-season influenza and COVID-19 vaccination and lifetime-reported receipt of pneumococcal, Tdap-polio, and herpes zoster vaccines showed distinct patterns and should not be interpreted as equivalent measures of schedule-concordant vaccination. An age-adjusted association between female sex and higher pneumococcal receipt was observed. Among self-described annual influenza recipients, more than one-fifth had not yet reported current-season influenza vaccination at the time of the survey.

The descriptive patterns observed in this study identify several potential target groups for future interventions. The observed age gradients for influenza and the COVID-19 booster suggest that younger and middle-aged patients with HF may represent potential target groups for future tailored interventions. The mid-to-older peak in pneumococcal uptake and the uniformly low herpes zoster uptake each define distinct gaps potentially amenable to vaccine-specific messaging. The low zoster uptake may be particularly relevant given observational associations between zoster vaccination and lower cardiovascular event rates [9]. Ever-reported pneumococcal receipt was higher in women in age-adjusted analyses. This finding may be compatible with previously described sex differences in preventive-care use [6,15], although the mechanisms underlying the association could not be assessed in our study. The higher pneumococcal uptake observed in women appeared to diminish with age (Table S5); however, given the multiplicity of exploratory comparisons and the attenuation of this interaction after adjustment for multiple testing, this finding should be considered hypothesis-generating and requires confirmation in an independent sample. Regional differences were observed in exploratory models adjusted for age and sex only. However, small and non-population-based regional samples and the absence of socioeconomic, health-care-access, clinical, and digital-engagement variables preclude attribution of these differences to geography itself. These findings should therefore be considered hypothesis-generating only. Although influenza uptake in this cohort was numerically higher than the 49.6% coverage reported among at-risk adults nationally for the 2025–2026 campaign [16], differences in population (telemonitored HF vs. all at-risk adults), ascertainment method (self-report vs. administrative records), and measurement timing preclude direct comparison; this figure is presented for context only and should not be interpreted as evidence that telemonitoring enrollment improves preventive engagement.

A further pattern emerged among the oldest patients. Those aged 85 years or older expressed particularly favorable attitudes toward vaccination, yet their ever-reported pneumococcal receipt was lower than in patients a decade younger. Grouping patients descriptively into younger than 65 years, 65 to 84 years, and 85 years or older confirmed that this decline was specific to the oldest group rather than a continuation of the trend observed across the broader population aged 65 years or older (ever-reported pneumococcal receipt, 29.0%, 38.7%, and 28.8%, respectively). Their interest in telemonitoring-based reminders was also the lowest of any age group. This dissociation between favorable attitudes and lower uptake coincided with a higher proportion of nurse-delivered influenza vaccination among the oldest patients, although the setting of vaccination, mobility status, and digital literacy were not recorded. Reported barriers to digital engagement among older adults may partly limit the effectiveness of application-based reminders in this group [17], although this was not directly assessed in our study. Although immune responses to vaccination may be attenuated with advancing age [5,18], the high absolute risk of infection and its complications makes vaccination particularly important in the oldest patients. These findings suggest that reminder strategies for the oldest patients may need to be evaluated using channels other than application-based prompts alone.

The vaccination pathways have operational implications. Community pharmacies were the dominant provider for most vaccines, consistent with the 2023 expansion authorizing pharmacists to prescribe and administer many vaccines in the adult schedule [19], whereas Tdap-polio—recommended at ages 25, 45, and 65 years and every 10 years thereafter [20]—remained predominantly GP-delivered. Telemonitoring could support pathway-specific prompts and shared documentation across vaccinators. More than half of participants expressed interest in receiving reminders. Previous evidence indicates that reminder-recall interventions can improve vaccination uptake, with SMS among effective single-channel approaches [21]. Whether SMS or in-app prompts improve vaccination uptake within HF telemonitoring requires prospective evaluation. A pragmatic randomized or stepped-wedge trial comparing in-app notifications, SMS reminders, and usual care, ideally linked to verified vaccination records, could evaluate the effectiveness of these strategies on vaccine uptake and clinical outcomes.

### Limitations

This study has several limitations. First, vaccination status and all measures were self-reported and were not verified against immunization records, so recall and social-desirability bias may have inflated self-reported uptake and the proportion reporting favorable attitudes. Second, respondents were enrolled in a telemonitoring program and were sufficiently digitally engaged to complete an electronic survey, which limits generalizability to all patients with HF and likely under-represents the least digitally engaged older patients. Because the questionnaire was administered only through the application, patients who interact with the telemonitoring program exclusively by telephone were not eligible and are not represented in this sample. The response rate was 22.7%, and characteristics of nonresponders were unavailable, precluding direct comparison between respondents and nonrespondents. Substantial nonresponse bias is therefore possible, particularly if vaccination behaviors, digital engagement, or preventive-care attitudes differed between these groups. The direction and magnitude of this potential bias cannot be determined from the available data. Third, the analytical dataset did not include HF phenotype, left ventricular ejection fraction, NYHA functional class, HF etiology, frailty, detailed comorbidities, recent hospitalization, or current HF therapy. Residual confounding is therefore substantial, and associations adjusted only for age and/or sex should not be interpreted as independent effects. Fourth, several regions had small samples, and regional estimates were adjusted only for age and sex. Because socioeconomic conditions, health care access, digital engagement, telemonitoring penetration, and other potential regional confounders were not available, these analyses should be considered exploratory. Moreover, the regional distribution of respondents was not population-based and should not be interpreted as nationally representative. Fifth, multiple comparisons were performed across vaccines, age strata, and regions; although the age-by-sex interaction tests were adjusted for multiplicity, other secondary and exploratory findings were not, and should be considered hypothesis-generating pending confirmation in an independent sample. Sixth, the survey was conducted from 5 November to 28 November 2025, during rather than after the 2025–2026 influenza and COVID-19 vaccination campaigns; current-season uptake may therefore underestimate final seasonal coverage, particularly for age groups that vaccinate later in the season, which should be considered when comparing uptake across strata. Seventh, lifetime self-reported receipt of the Tdap-polio booster (“ever”) cannot be interpreted as adherence to the age-specific French booster schedule, and the setting of vaccine administration and patients’ mobility or living arrangements were not recorded. Individual vaccine indications, contraindications, prior adverse reactions, and current schedule eligibility were not assessed. Consequently, non-reported vaccine receipt cannot be equated with inappropriate non-vaccination or with a definite missed vaccination opportunity. Similarly, lifetime self-reported pneumococcal vaccination did not distinguish vaccine type, timing, or completion of the currently recommended schedule. Finally, the cross-sectional design cannot establish whether telemonitoring-based reminder interventions improve vaccination uptake or clinical outcomes.

## 5. Conclusions

Among digitally connected patients with HF enrolled in a nationwide telemonitoring program, vaccination attitudes were generally favorable. Current-season vaccination and lifetime-reported vaccine receipt showed distinct age patterns, and an age-adjusted association between female sex and higher pneumococcal receipt was observed. Community pharmacies were central to vaccine delivery. These descriptive findings identify potential target groups for future telemonitoring-based preventive interventions; whether such interventions improve vaccination uptake or clinical outcomes requires prospective evaluation.

## Figures and Tables

**Table 1:** Participant Characteristics, Perceived Vaccination Knowledge, and Attitudes (N = 1100).

Characteristic	No. (%) [Or as Noted]
Age, mean (SD), years	71.5 (12.6)
Age, median [range], years	73 [25–100]
**Age group, y**	
18–49	60 (5.5)
50–64	250 (22.7)
65–74	298 (27.1)
75–84	322 (29.3)
≥85	170 (15.5)
**Sex**	
Men	718 (65.3)
Women	379 (34.5)
Not specified	3 (0.3)
**Perceived knowledge and attitudes**	
Considers vaccination useful	963 (87.5)
Knows where to be vaccinated	1056 (96.0)
Reports knowing age-recommended vaccines	825 (75.0)
Would vaccinate if physician recommended	882 (80.2)
Wants reminders from telemonitoring team	606 (55.1)
Reported prior difficulty accessing vaccination	19 (1.7)

Abbreviation: SD, standard deviation.

**Table 2:** Self-Reported Vaccine Receipt Overall and by Sex.

Vaccine	Overall, % (95% CI)	Women, No. (%)	Men, No. (%)	Age-Adjusted OR (95% CI); *p*-Value
Influenza (2025–2026 season)	62.2 (59.3–65.0)	235 (62.0)	447 (62.3)	0.98 (0.75–1.27); *p* = 0.85
COVID-19 booster (2025–2026 season)	31.4 (28.7–34.2)	108 (28.5)	236 (32.9)	0.79 (0.60–1.04); *p* = 0.09
Pneumococcal (ever received)	34.5 (31.7–37.3)	160 (42.2)	218 (30.4)	1.67 (1.29–2.17); *p* < 0.001
Tdap-polio booster (ever received)	38.5 (35.7–41.5)	152 (40.1)	270 (37.6)	1.12 (0.86–1.44); *p* = 0.40
Herpes zoster (ever received)	11.5 (9.8–13.6)	53 (14.0)	73 (10.2)	1.42 (0.97–2.07); *p* = 0.07

Abbreviations: CI—confidence interval; OR—odds ratio; Tdap-polio—tetanus–diphtheria–pertussis–poliomyelitis. Odds ratios compare women with men and are adjusted for age as a continuous variable. Overall denominators include all 1100 respondents; sex-specific percentages exclude 3 respondents with unspecified sex. Confidence intervals were calculated using the Wilson method. In the primary analysis, “No” and “I don’t know” responses were both included in the denominator; results excluding “I don’t know” responses are presented in Table S8.

**Table 3:** Vaccinating Provider Among Vaccinated Respondents, by Vaccine.

Vaccine	Vaccinated, No.	Pharmacy, No. (%)	General Practitioner, No. (%)	Nurse, No. (%)	Other Provider, No. (%)
Influenza (2025–2026 season)	684	454 (66.4)	54 (7.9)	169 (24.7)	7 (1.0)
COVID-19 booster (2025–2026 season)	345	280 (81.2)	14 (4.1)	44 (12.8)	7 (2.0)
Pneumococcal (ever received)	379	163 (43.0)	113 (29.8)	91 (24.0)	12 (3.2)
Tdap-polio booster (ever received)	424	85 (20.0)	230 (54.2)	80 (18.9)	29 (6.8)
Herpes zoster (ever received)	127	75 (59.1)	27 (21.3)	23 (18.1)	2 (1.6)

Percentages are among respondents reporting receipt of each vaccine and may not total 100 because of rounding. The “Other provider” category combines vaccination centers, geriatricians, and cardiologists. Provider distributions differed significantly by sex for influenza (*p* = 0.03) and the COVID-19 booster (*p* = 0.004).

## Data Availability

The data presented in this study are not publicly available because of institutional and regulatory restrictions relating to pseudonymized health data. Reasonable requests may be addressed to the corresponding author and will be considered subject to institutional authorization and applicable data-protection requirements.

## Supplementary Materials

The following supporting information can be downloaded at: https://www.mdpi.com/article/10.3390/jcm15176933/s1, This supplement contains additional analyses referenced in the main text: self-reported seasonal vaccination and lifetime vaccine receipt by five age strata (Table S1); Self-reported seasonal vaccination and lifetime vaccine receipt by three broad age groups (Table S2); Perceived vaccination knowledge and attitudes by age group (Table S3); Restricted Cubic Spline Models of Self-Reported Seasonal Vaccination and Lifetime Vaccine Receipt by Continuous Age and Predicted Self-Reported Seasonal Vaccination and Lifetime Vaccine Receipt by Continuous Age, With 95% Confidence Intervals (Table S4 and Figure S1); Exploratory Age-by-Sex Interaction Models, by Vaccine (Table S5); Vaccinating Provider for Seasonal Influenza, by Age Group (Table S6); Self-Reported Seasonal Vaccination and Lifetime Pneumococcal Vaccine Receipt by French Region (Table S7); Distribution of Vaccination-Status Responses and Sensitivity Estimates Excluding “I Don’t Know” Responses and Sensitivity Analyses of Age and Sex Associations After Exclusion of “I Don’t Know” Responses (Tables S8 and S9); Sensitivity Analysis of Regional Heterogeneity Restricted to Regions With at Least 20 Respondents (Table S10); and the patient questionnaire Satelia Cardio Vaccination Survey Questionnaire (Questionnaire S1).
