# Supplementary material for: Perceived Vaccination Knowledge and Self-Reported Uptake in a Nationwide Heart Failure Telemonitoring Cohort in France
Source: J Clin Med. 2026 Sep 7;15(17):6933. doi: 10.3390/jcm15176933 (PMC13566655; doi:10.3390/jcm15176933)
Supplement: Supplementary file 1 [file jcm-15-06933-s001.zip › jcm-4490383-supplementary.pdf]

Supplementary Online Content

This supplement contains additional analyses referenced in the main text: self-reported seasonal vaccination and lifetime vaccine receipt by 5 age strata (Table S1); a descriptive comparison across 3 broad age groups (Table S2); perceived vaccination knowledge, attitudes, and reminder interest by age group (Table S3); results of the restricted cubic spline models of self-reported seasonal vaccination and lifetime vaccine receipt by continuous age (Table S4 and Figure S1); exploratory age-by-sex interaction models (Table S5); the distribution of vaccinating provider for influenza across the 5 age strata (Table S6); self-reported seasonal vaccination and lifetime pneumococcal vaccine receipt by French region (Table S7); the distribution of "I don't know" responses and vaccine receipt after their exclusion (Table S8); corresponding sensitivity analyses of age and sex associations (Table S9); a sensitivity analysis of regional heterogeneity restricted to regions with at least 20 respondents (Table S10); and the patient questionnaire (Questionnaire S1).

This supplementary material has been provided by the authors to give readers additional information about their work.

Table S1. Self-reported seasonal vaccination and lifetime vaccine receipt by five age strata

Age group, y (No.)	Influenza, No./total (%)	COVID-19 booster, No./total (%)	Pneumococcal, No./total (%)	Tdap-polio booster, No./total (%)	Herpes zoster, No./total (%)
18-49 (n = 60)	21/60 (35.0) *95% CI, 24.2-47.6*	5/60 (8.3) *95% CI, 3.6-18.1*	12/60 (20.0) *95% CI, 11.8-31.8*	26/60 (43.3) *95% CI, 31.6-55.9*	3/60 (5.0) *95% CI, 1.7-13.7*
50-64 (n = 250)	132/250 (52.8) *95% CI, 46.6-58.9*	44/250 (17.6) *95% CI, 13.4-22.8*	78/250 (31.2) *95% CI, 25.8-37.2*	101/250 (40.4) *95% CI, 34.5-46.6*	8/250 (3.2) *95% CI, 1.6-6.2*
65-74 (n = 298)	192/298 (64.4) *95% CI, 58.8-69.6*	102/298 (34.2) *95% CI, 29.1-39.8*	129/298 (43.3) *95% CI, 37.8-49.0*	124/298 (41.6) *95% CI, 36.2-47.3*	42/298 (14.1) *95% CI, 10.6-18.5*
75-84 (n = 322)	217/322 (67.4) *95% CI, 62.1-72.3*	122/322 (37.9) *95% CI, 32.8-43.3*	111/322 (34.5) *95% CI, 29.5-39.8*	113/322 (35.1) *95% CI, 30.1-40.5*	49/322 (15.2) *95% CI, 11.7-19.5*
≥85 (n = 170)	122/170 (71.8) *95% CI, 64.6-78.0*	72/170 (42.4) *95% CI, 35.2-49.9*	49/170 (28.8) *95% CI, 22.5-36.0*	60/170 (35.3) *95% CI, 28.5-42.7*	25/170 (14.7) *95% CI, 10.2-20.8*

Percentages are among respondents in each age group (N = 1100 overall), with 95% CIs by the Wilson method. Influenza and the COVID-19 booster refer to the 2025-2026 season; pneumococcal, Tdap-polio booster, and herpes zoster refer to whether participants had ever received the vaccine. See Figure 1 for the corresponding bar chart.

Table S2. Self-reported seasonal vaccination and lifetime vaccine receipt by three broad age groups

Age group, y (No.)	Influenza, % (95% CI)	COVID-19 booster, % (95% CI)	Pneumococcal, % (95% CI)	Tdap-polio booster, % (95% CI)	Herpes zoster, % (95% CI)
<65 (n = 310)	49.4 (43.8-54.9)	15.8 (12.2-20.3)	29.0 (24.3-34.3)	41.0 (35.6-46.5)	3.5 (2.0-6.2)
65-84 (n = 620)	66.0 (62.2-69.6)	36.1 (32.4-40.0)	38.7 (35.0-42.6)	38.2 (34.5-42.1)	14.7 (12.1-17.7)
≥85 (n = 170)	71.8 (64.6-78.0)	42.4 (35.2-49.9)	28.8 (22.5-36.0)	35.3 (28.5-42.7)	14.7 (10.2-20.8)

Percentages are among respondents in each broad age group (N = 1100 overall), with 95% CIs by the Wilson method. Influenza and the COVID-19 booster refer to the 2025-2026 season; pneumococcal, Tdap-polio booster, and herpes zoster refer to whether participants had ever received the vaccine. This descriptive 3-group comparison (<65, 65-84, and ≥85 years) complements the 5-stratum description in Table S1 and the continuous-age spline models in Table S4 and Figure S1.

Table S3. Perceived vaccination knowledge and attitudes by age group

Age group, y (No.)	Considers vaccination useful, %	Reports knowing age-recommended vaccines, %	Knows where to be vaccinated, %	Prior difficulty accessing vaccination, %	Would vaccinate if physician recommended, %	Wants reminders from telemonitoring team, %
18-49 (n = 60)	78.3	40.0	85.0	5.0	55.0	61.7
50-64 (n = 250)	83.6	68.0	94.4	2.8	71.6	54.8
65-74 (n = 298)	87.9	80.2	96.6	1.3	82.2	54.4
75-84 (n = 322)	89.1	80.1	97.5	1.6	85.7	58.7
≥85 (n = 170)	92.9	78.8	98.2	0.0	87.6	47.6

Percentages are among respondents in each age group (N = 1100 overall). See Table 1 for the corresponding overall (all-ages) percentages.

Table S4. Restricted Cubic Spline Models of Self-Reported Seasonal Vaccination and Lifetime Vaccine Receipt by Continuous Age

Vaccine	P value, overall association with age	P value, nonlinearity	Shape of the age–uptake relationship (sex-adjusted spline model)
Seasonal influenza	< 0.001	0.96	Monotonic increase across the age range (24% at 25 y to 80% at 100 y); no evidence of nonlinearity
COVID-19 booster	< 0.001	0.04	Declined from young adulthood to a nadir near age 47 y (8.6%), then rose progressively with age
Pneumococcal	0.03	0.02	Peaked at age 71 y (39.9%; 95% CI, 35.1%-44.9%), then declined toward the oldest ages
Tdap-polio booster	0.47	0.77	No significant association with age; probability remained comparatively stable (roughly 35%-44%) across the age range
Herpes zoster	< 0.001	0.001	Remained low before approximately 65 y and rose thereafter, plateauing around 80-85 y

Models were adjusted for sex but not region. Overall association and nonlinearity were assessed using likelihood-ratio tests. P values were not adjusted for multiple testing across the 5 vaccines. Predicted percentages were marginally standardized over the observed sex distribution. See Figure S1 for the corresponding predicted probability curves with 95% CIs. Influenza and COVID-19 booster outcomes refer to self-reported vaccination during the 2025–2026 season; pneumococcal, Tdap-polio, and herpes zoster outcomes refer to lifetime self-reported receipt and do not indicate schedule-concordant vaccination.

Figure S1. Predicted Self-Reported Seasonal Vaccination and Lifetime Vaccine Receipt by Continuous Age, With 95% Confidence Intervals

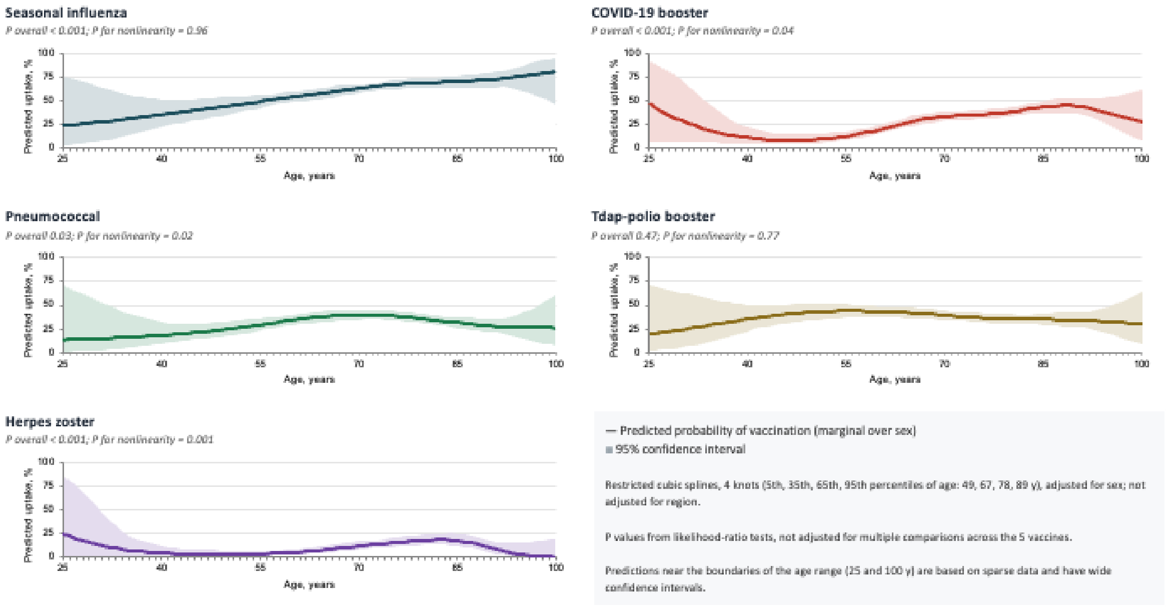

Restricted cubic spline estimates of self-reported seasonal vaccination and lifetime vaccine receipt by continuous age, with 95% confidence intervals. Models used 4 knots at the 5th, 35th, 65th, and 95th percentiles of age (49, 67, 78, and 89 years), were adjusted for sex, and were not adjusted for region. Shaded areas represent 95% confidence intervals calculated using the delta method. Predictions near the boundaries of the observed age range (25 and 100 years) are based on sparse data and should be interpreted cautiously. Influenza and COVID-19 booster outcomes refer to self-reported vaccination during the 2025–2026 season; pneumococcal, Tdap-polio, and herpes zoster outcomes refer to lifetime self-reported receipt and do not indicate schedule-concordant vaccination.

Table S5. Exploratory Age-by-Sex Interaction Models, by Vaccine

Vaccine	Interaction coefficient (per year)	Interaction OR (per year)	95% CI	Nominal P value	Benjamini–Hochberg-adjusted P value
Seasonal influenza	0.0060	1.006	0.985-1.027	0.58	0.96
COVID-19 booster	-0.0019	0.998	0.975-1.022	0.87	0.99
Pneumococcal	-0.0243	0.976	0.956-0.996	0.02	0.06
Tdap-polio booster	-0.0229	0.977	0.958-0.997	0.03	0.06
Herpes zoster	0.0003	1.000	0.968-1.033	0.99	0.99

Interaction coefficients, odds ratios (ORs), and 95% CIs are for the continuous age-by-female interaction term in a logistic model of the form outcome ~ female + age + female×age. P values were adjusted for multiplicity across the 5 tests using the Benjamini–Hochberg procedure. These analyses are exploratory and hypothesis-generating. Influenza and COVID-19 booster outcomes refer to self-reported vaccination during the 2025–2026 season; pneumococcal, Tdap-polio, and herpes zoster outcomes refer to lifetime self-reported receipt and do not indicate schedule-concordant vaccination.

Table S6. Vaccinating Provider for Seasonal Influenza, by Age Group

Age group, y	No. vaccinated	Pharmacy, %	Nurse, %	General practitioner, %	Vaccination center, %	Other provider, %
18-49	21	47.6	23.8	28.6	0.0	0.0
50-64	132	74.2	15.9	7.6	2.3	0.0
65-74	192	70.3	19.8	9.4	0.0	0.5
75-84	217	65.9	29.5	3.7	0.0	0.9
≥85	122	55.7	33.6	9.8	0.8	0.0

Percentages are among respondents in each age group who reported receipt of the seasonal influenza vaccine (n = 684 overall). The 'Other provider' category combines geriatricians and cardiologists. Percentages may not total 100 because of rounding.

Table S7. Self-Reported Seasonal Vaccination and Lifetime Pneumococcal Vaccine Receipt by French Region

Region	No. of respondents	Influenza, No./total (%)	COVID-19 booster, No./total (%)	Pneumococcal, No./total (%)
Auvergne-Rhône-Alpes	79	55/79 (69.6)<br>*95% CI, 58.8-78.7*	30/79 (38.0)<br>*95% CI, 28.1-49.0*	36/79 (45.6)<br>*95% CI, 35.0-56.5*
Bourgogne-Franche-Comté	83	54/83 (65.1)<br>*95% CI, 54.3-74.4*	21/83 (25.3)<br>*95% CI, 17.2-35.6*	24/83 (28.9)<br>*95% CI, 20.3-39.4*
Centre-Val de Loire	57	41/57 (71.9)<br>*95% CI, 59.2-81.9*	24/57 (42.1)<br>*95% CI, 30.2-55.0*	16/57 (28.1)<br>*95% CI, 18.1-40.8*
Grand Est	133	69/133 (51.9)<br>*95% CI, 43.5-60.2*	30/133 (22.6)<br>*95% CI, 16.3-30.4*	58/133 (43.6)<br>*95% CI, 35.5-52.1*
Guadeloupe	11	3/11 (27.3)<br>*95% CI, 9.7-56.6*	2/11 (18.2)<br>*95% CI, 5.1-47.7*	3/11 (27.3)<br>*95% CI, 9.7-56.6*
Hauts-de-France	97	62/97 (63.9)<br>*95% CI, 54.0-72.8*	29/97 (29.9)<br>*95% CI, 21.7-39.6*	23/97 (23.7)<br>*95% CI, 16.4-33.1*
Île-de-France	173	120/173 (69.4)<br>*95% CI, 62.1-75.8*	70/173 (40.5)<br>*95% CI, 33.4-47.9*	45/173 (26.0)<br>*95% CI, 20.0-33.0*
La Réunion	13	8/13 (61.5)<br>*95% CI, 35.5-82.3*	2/13 (15.4)<br>*95% CI, 4.3-42.2*	1/13 (7.7)<br>*95% CI, 1.4-33.3*
Martinique	15	1/15 (6.7)<br>*95% CI, 1.2-29.8*	1/15 (6.7)<br>*95% CI, 1.2-29.8*	1/15 (6.7)<br>*95% CI, 1.2-29.8*
Normandie	43	28/43 (65.1)<br>*95% CI, 50.2-77.6*	18/43 (41.9)<br>*95% CI, 28.4-56.7*	16/43 (37.2)<br>*95% CI, 24.4-52.1*
Nouvelle-Aquitaine	204	122/204 (59.8)<br>*95% CI, 53.0-66.3*	62/204 (30.4)<br>*95% CI, 24.5-37.0*	66/204 (32.4)<br>*95% CI, 26.3-39.0*
Occitanie	87	50/87 (57.5)<br>*95% CI, 47.0-67.3*	26/87 (29.9)<br>*95% CI, 21.3-40.2*	38/87 (43.7)<br>*95% CI, 33.7-54.1*
Pays de la Loire	22	10/22 (45.5)<br>*95% CI, 26.9-65.3*	5/22 (22.7)<br>*95% CI, 10.1-43.4*	8/22 (36.4)<br>*95% CI, 19.7-57.0*
Provence-Alpes-Côte d'Azur	77	57/77 (74.0)<br>*95% CI, 63.3-82.5*	23/77 (29.9)<br>*95% CI, 20.8-40.8*	41/77 (53.2)<br>*95% CI, 42.2-64.0*

Regions with fewer than 10 respondents are not shown. Influenza and the COVID-19 booster refer to the 2025-2026 season; pneumococcal vaccination refers to whether participants had ever received the vaccine. Confidence intervals were calculated using the Wilson method. Regional comparisons were exploratory because sample sizes were small in several regions and analyses were adjusted only for age and sex. See Figure 2 for the corresponding bar chart.

Table S8. Distribution of Vaccination-Status Responses and Sensitivity Estimates Excluding "I Don't Know" Responses

Vaccine	Yes, No. (%)	No, No. (%)	I don't know, No. (%)	Primary estimate, % (95% CI)	Sensitivity estimate, % (95% CI)	Absolute difference, percentage points
Seasonal influenza (2025–2026 season)	684 (62.2)	406 (36.9)	10 (0.9)	62.2 *95% CI, 59.3–65.0*	62.8 *95% CI, 59.8–65.6*	+0.6
COVID-19 booster (2025–2026 season)	345 (31.4)	745 (67.7)	10 (0.9)	31.4 *95% CI, 28.7–34.2*	31.7 *95% CI, 29.0–34.5*	+0.3
Pneumococcal vaccine (ever received)	379 (34.5)	619 (56.3)	102 (9.3)	34.5 *95% CI, 31.7–37.3*	38.0 *95% CI, 35.0–41.0*	+3.5
Tdap-polio booster (ever received)	424 (38.5)	512 (46.5)	164 (14.9)	38.5 *95% CI, 35.7–41.5*	45.3 *95% CI, 42.1–48.5*	+6.8
Herpes zoster vaccine (ever received)	127 (11.5)	903 (82.1)	70 (6.4)	11.5 *95% CI, 9.8–13.6*	12.3 *95% CI, 10.5–14.5*	+0.8

No. denotes number. In the primary analysis, "No" and "I don't know" responses were both included in the denominator. In the sensitivity analysis, "I don't know" responses were excluded and vaccine receipt was calculated among respondents providing a definite "Yes" or "No" response. Confidence intervals were calculated using the Wilson method.

Table S9. Sensitivity Analyses of Age and Sex Associations After Exclusion of "I Don't Know" Responses

Vaccine	Definite Yes/No responses, No.	P value for overall age association	P value for nonlinearity	Women vs men, age-adjusted OR (95% CI)	P value	Comparison with primary analysis
Seasonal influenza	1090	< 0.001	0.68	0.98 (0.75–1.28)	0.88	The overall age association and sex association were materially unchanged; there was no evidence of nonlinearity in either analysis.
COVID-19 booster	1090	< 0.001	0.10	0.78 (0.59–1.03)	0.08	The overall age association and sex association were materially unchanged; evidence of nonlinearity was attenuated from P = 0.04 to P = 0.10.
Pneumococcal	998	0.04	0.01	1.62 (1.24–2.11)	< 0.001	The overall age association, evidence of nonlinearity, and sex association were materially unchanged.
Tdap-polio booster	936	0.02	0.76	1.05 (0.80–1.38)	0.71	The sex association and absence of nonlinearity were materially unchanged; an overall age association emerged after exclusion of 'I don't know' responses (P = 0.02 vs P = 0.47).

Vaccine	Definite Yes/No responses, No.	P value for overall age association	P value for nonlinearity	Women vs men, age-adjusted OR (95% CI)	P value	Comparison with primary analysis
Herpes zoster	1030	< 0.001	< 0.001	1.42 (0.97–2.09)	0.07	The overall age association, evidence of nonlinearity, and sex association were materially unchanged.

"I don't know" responses were excluded separately for each vaccine. Age associations were estimated using restricted cubic splines with 4 knots at the 5th, 35th, 65th, and 95th percentiles of age (49, 67, 78, and 89 years), adjusted for sex. Odds ratios compare women with men and are adjusted for age as a continuous variable. These analyses should be interpreted as sensitivity analyses of the primary findings reported in Table 2 and Table S4. Counts of definite Yes/No responses are descriptive denominators. Respondents with unspecified sex were excluded from the sex-adjusted spline and logistic regression models. Influenza and COVID-19 booster outcomes refer to self-reported vaccination during the 2025–2026 season; pneumococcal, Tdap-polio, and herpes zoster outcomes refer to lifetime self-reported receipt and do not indicate schedule-concordant vaccination.

Table S10. Sensitivity Analysis of Regional Heterogeneity Restricted to Regions With at Least 20 Respondents

Vaccine	Primary regional sample (14 regions; n = 1094), P value	Restricted regional sample (11 regions; n = 1055), P value	Regional heterogeneity in the sensitivity analysis
Seasonal influenza	< 0.001	0.04	Remained
COVID-19 booster	0.02	0.03	Remained
Pneumococcal	< 0.001	< 0.001	Remained
Tdap-polio booster	0.86	0.87	Remained absent
Herpes zoster	0.26	0.49	Remained absent

Regions with fewer than 20 respondents (Guadeloupe, n = 11; La Réunion, n = 13; Martinique, n = 15) were excluded from the sensitivity analysis. Regional heterogeneity was tested by likelihood-ratio tests comparing logistic models with and without region, adjusting for age and sex, among respondents with known sex. The primary analysis included all 14 regions with at least 10 respondents. Consistent with the primary analysis, evidence of regional heterogeneity remained for influenza, the COVID-19 booster, and pneumococcal vaccination, and remained absent for the Tdap-polio booster and herpes zoster vaccination. The sample sizes shown are descriptive regional sample sizes. Model-specific analytical sample sizes were slightly smaller because respondents with unspecified sex were excluded from adjusted analyses.

Questionnaire S1. Satelia Cardio Vaccination Survey Questionnaire—English Translation

This questionnaire has been translated into English from the original French instrument administered electronically through the Satelia telemonitoring application in November 2025. Question wording matches the original French items; answer choices reflect the response options recorded in the data. The "Who vaccinated you?" answer list is presented as a single consolidated set of options, since the same option list was reused across the 5 vaccine-specific follow-up questions. Original French wording is provided in brackets for traceability.

A. Respondent Information

Q1. What is your age?
[Quel est votre âge ?]

Answer: _____ years

Q2. What is your postal code?
[Quel est votre code postal ?]

Answer: ______________

Q3. You are:
[Vous êtes :]
☐ A woman ☐ A man ☐ I prefer not to specify

B. Vaccination Knowledge and Attitudes

Q4. Do you know which vaccinations are recommended for your age?
[Savez-vous quelles sont les vaccinations recommandées pour votre âge ?]
☐ Yes ☐ No ☐ A little

Q5. Do you think vaccination is useful to protect your health?
[Pensez-vous que la vaccination est utile pour protéger votre santé ?]
☐ Yes ☐ I don't know ☐ No

Q6. Do you know where you can get vaccinated (physician, pharmacy, center…)?
[Savez-vous où vous pouvez recevoir vos vaccins (médecin, pharmacie, centre…) ?]
☐ Yes ☐ No ☐ I don't know

Q7. Have you ever encountered difficulties getting vaccinated?
[Avez-vous déjà rencontré des difficultés pour vous faire vacciner ?]
☐ No ☐ Yes

Q8. Would you be interested in Satelia® or your care team reminding you which vaccines are due?
[Seriez-vous intéressé(e) que Satelia® ou votre équipe soignante vous rappelle les vaccins à faire ?]
☐ Yes ☐ No

Q9. If a vaccine is recommended by your physician, do you intend to get it?
[Si un vaccin vous est recommandé par votre médecin, avez-vous l'intention de le faire ?]
☐ Yes ☐ I don't know ☐ No

C. Vaccine-Specific Questions

C1. Seasonal Influenza

Q10. Do you get the influenza vaccine every year?
[Faites-vous chaque année la vaccination contre la grippe ?]
☐ Yes ☐ No ☐ Not systematically

Q11. Have you received the seasonal influenza vaccine this year?
[Avez-vous reçu le vaccin contre la grippe saisonnière cette année ?]
☐ Yes ☐ No ☐ I don't know

Q12. Who vaccinated you?

[Qui vous a vacciné ?]
↳ *asked only if Q11 = Yes*
☐ Primary care physician ☐ Pharmacist ☐ Nurse ☐ Vaccination center ☐ Geriatrician ☐ Cardiologist

C2. COVID-19

Q13. Do you get the COVID-19 vaccine every year?
[Faites-vous chaque année la vaccination contre le COVID-19 ?]
☐ Yes ☐ No ☐ Not systematically

Q14. Have you received the COVID-19 vaccine this year?
[Avez-vous reçu le vaccin contre le COVID-19, cette année ?]
☐ Yes ☐ No ☐ I don't know

Q15. Who vaccinated you?
[Qui vous a vacciné ?]
↳ *asked only if Q14 = Yes*
☐ Primary care physician ☐ Pharmacist ☐ Nurse ☐ Vaccination center ☐ Geriatrician ☐ Cardiologist

C3. Pneumococcal

Q16. Have you received the pneumococcal vaccine?
[Avez-vous reçu le vaccin contre le pneumocoque ?]
☐ Yes ☐ No ☐ I don't know

Q17. Who vaccinated you?
[Qui vous a vacciné ?]
↳ *asked only if Q16 = Yes*
☐ Primary care physician ☐ Pharmacist ☐ Nurse ☐ Vaccination center ☐ Geriatrician ☐ Cardiologist

C4. Tetanus–Diphtheria–Pertussis–Poliomyelitis (Tdap-Polio)

Q18. Have you received the Tdap-polio vaccine (diphtheria/tetanus/poliomyelitis/pertussis)?
[Avez-vous reçu le vaccin contre le DTPC (diphtérie/tétanos/poliomyélite/coqueluche) ?]
☐ Yes ☐ No ☐ I don't know

Q19. Who vaccinated you?
[Qui vous a vacciné ?]
↳ *asked only if Q18 = Yes*
☐ Primary care physician ☐ Pharmacist ☐ Nurse ☐ Vaccination center ☐ Geriatrician ☐ Cardiologist

C5. Herpes Zoster (Shingles)

Q20. Have you received the herpes zoster (shingles) vaccine?
[Avez-vous reçu le vaccin contre le zona ?]
☐ Yes ☐ No ☐ I don't know

Q21. Who vaccinated you?
[Qui vous a vacciné ?]
↳ *asked only if Q20 = Yes*
☐ Primary care physician ☐ Pharmacist ☐ Nurse ☐ Vaccination center ☐ Geriatrician ☐ Cardiologist

---

A submission timestamp ("Horodateur") was recorded automatically for each response and is not a questionnaire item.
